# Beyond cortisol: evaluating serotonin, brain-derived neurotrophic factor, and oxytocin as indicators of equine welfare across three training regimens

**DOI:** 10.1186/s12917-026-05507-7

**Published:** 2026-04-30

**Authors:** Maciej Kacprzyk, Dawid Tobolski, Paula Kiełbik, Jowita Grzędzicka-Agko, Dominika Milczek-Haduch, Elżbieta Stefanik, Izabela Dąbrowska, Bartosz Pawliński, Marcin Gołębiewski, Olga Witkowska-Piłaszewicz

**Affiliations:** 1 Institute of Animal Sciences, Department of Animal Breeding, Warsaw University of Life Sciences (SGGW), Nowoursynowska 166, Warsaw, 02-787 Poland; 2 Institute of Veterinary Medicine, Department of Large Animal Diseases and Clinic, Warsaw University of Life Sciences (SGGW), Nowoursynowska 166, Warsaw, 02-787 Poland

**Keywords:** Equine welfare, Exercise stress, Serotonin, Brain-derived neurotrophic factor, Oxytocin, Cortisol, Show jumping, Racehorses, Leisure horses

## Abstract

**Background:**

Quantifying the balance between workload, stress, and recovery across equine disciplines remains challenging, and no single circulating biomarker provides a definitive index of welfare-related stress load. Cortisol, oxytocin, serotonin (5-hydroxytryptamine, 5-HT), and brain-derived neurotrophic factor (BDNF) may provide complementary information on endocrine and neuromodulatory responses.

**Methods:**

Paired serum samples were collected from show jumping horses (*n* = 17) before and after competition, racehorses (*n* = 43 enrolled, *n* = 41 analyzed) before and after sessions at season start (T1), mid-season (T2), and race day (R), and leisure horses (*n* = 14) at rest before and after the riding season. Biomarkers were quantified by ELISA. Changes were modeled as the natural logarithm of the post/pre ratio [ln(post/pre)] using intercept-only linear models (show jumping, leisure) and linear mixed-effects models for racehorses with horse as a random intercept and session, sex, and breed as fixed effects.

**Results:**

In show jumping horses, 5-HT decreased after competition (post/pre ratio 0.634, 95% CI 0.490–0.820, *p* = 0.002). Breed-adjusted estimates supported a 5-HT decrease in mares (0.470, *p* = 0.026) and a BDNF increase in geldings (1.727, *p* = 0.021). In racehorses, 5-HT increased on race day in mares (1.948, 95% CI 1.258–3.015, *p* = 0.003) and stallions (2.207, *p* < 0.001) and did not differ from a post/pre ratio of 1.0 after T1 or T2. BDNF and oxytocin remained close to a post/pre ratio of 1.0 across race sessions (all *p* ≥ 0.507). Cortisol showed no significant session-specific changes, with lower post/pre ratios in stallions that did not reach statistical significance during T2 and race day (*p* = 0.059 and *p* = 0.064). Leisure horses showed no significant changes across the riding season for any marker (all *p* ≥ 0.166).

**Conclusions:**

Serotonin exhibited the strongest discipline-dependent responsiveness, with opposite directions of change after show jumping competition and on race day. BDNF responses were restricted to show jumping geldings, whereas oxytocin and cortisol were largely unchanged under the sampling schedule used. Under the applied sampling framework, multi-marker profiling may help characterize discipline-dependent neuroendocrine dynamics, but it does not by itself establish welfare status.

**Supplementary Information:**

The online version contains supplementary material available at 10.1186/s12917-026-05507-7.

## Introduction

Interest in equine welfare and stress responses has increased, driven by both performance expectations and ethical considerations [1–4]. Training and competition expose horses to physiological and psychological stressors, and stress load depends on exercise intensity and duration, fitness status, and competitive context. Limitations in owner and trainer knowledge can inadvertently compromise welfare-related states [5]. Inter-individual differences in stress reactivity further support individualized training and husbandry. Beneficial, appropriately dosed stress (eustress) can promote adaptation and performance [6]. In contrast, chronic stress is associated with impaired performance [7], suppressed immune function [8], and increased risk of gastric ulceration and welfare-relevant illness and injury in sport disciplines [9, 10]. Chronic stress is also associated with behavioral changes that can hinder learning [11], environmental coping [12], and overall well-being [13].

Given these multi-system consequences of chronic stress, no single biomarker provides a definitive measure of stress load, and welfare assessment is strengthened by integrating endocrine, physiological, and behavioral indicators. Physiological indices commonly include heart rate (HR), heart rate variability (HRV), respiratory rate, and ocular temperature measured by infrared thermography (IRT) [14, 15]. Behavioral observations, including tension-related behaviors, avoidance behaviors, and stereotypies, remain essential for interpreting physiological measures in context [16]. Among endocrine markers, cortisol is the most extensively studied stress-associated hormone in horses and humans and can be quantified in blood, saliva, urine, hair, and feces [17–20]. Importantly, cortisol exhibits both circadian and seasonal variation, with concentrations influenced by time of day as well as environmental factors such as photoperiod, temperature, and management conditions [17–20].

In response to acute stress, cortisol concentrations typically increase in humans and animals [21, 22] and support exercise-associated gluconeogenesis, amino acid mobilization, and lipolysis [23, 24]. In sport horses, training and competition have been associated with altered cortisol dynamics [18, 25]. Consequently, cortisol is increasingly evaluated alongside additional biomarkers discussed in welfare and performance research, including oxytocin, brain-derived neurotrophic factor (BDNF), and serotonin. Oxytocin has received attention as a potential welfare-related states indicator through roles in social bonding and stress modulation [26]. Lower cortisol concentrations and greater relaxation have been reported after oxytocin-associated interventions [27]. In horses, plasma oxytocin has been associated with docility, friendliness, and trainability [28, 29].

Serotonin (5-hydroxytryptamine, 5-HT) influences mood, fear responses, aggression, and sociability [30]. Welfare-linked alterations in serotonergic activity have been reported across species, including pigs, cattle, and horses [20, 31]. Improved cattle welfare associated with daily grazing has been linked to higher hair and plasma serotonin [32], whereas evidence in horses remains limited. BDNF, a neurotrophin involved in neuroplasticity and stress adaptation [33], is reduced in chronic stress states and several mental disorders [34, 35]. Equine data remain scarce, and technical constraints have limited reporting in at least one prior study [11].

Neuroendocrine pathways linking cortisol, oxytocin, BDNF, and serotonin are biologically interconnected, and joint evaluation of multiple biomarkers can provide a more comprehensive characterization of physiological and affective state than single-analyte interpretation [11]. In the current study, paired pre- to post-exposure changes in serum cortisol, oxytocin, BDNF, and 5-HT were quantified across three equine contexts: show jumping competition, race training and racing, and leisure riding across the riding season. The objective was to evaluate discipline- and sex-associated neuroendocrine patterns in relation to differences in workload and emotional and cognitive demands.

Based on the biological roles of serotonin, BDNF, oxytocin, and cortisol, and on differences in workload, emotional arousal, and cognitive demands between disciplines, we formulated the following hypotheses: (H1) serotonergic responses to activity would differ by discipline, with direction and magnitude of change reflecting competition context rather than exercise alone; (H2) BDNF responses would be more evident in disciplines characterized by high technical and cognitive demands, such as show jumping; and (H3) oxytocin and cortisol responses would show limited or sex-dependent changes under the applied sampling scheme.

## Materials and methods

### Ethics and regulatory compliance

All sampling procedures were performed as part of routine health monitoring and fitness assessment and did not involve experimental interventions. In accordance with European Directive 2010/63/EU and relevant national regulations, these procedures were classified as non-experimental clinical veterinary practices and did not require ethical approval. All procedures were conducted in accordance with applicable guidelines and reporting standards, including ARRIVE. Informed consent to participate from owners was obtained.

### Study design

This observational study evaluated circulating biomarkers in privately owned domestic horses (*Equus caballus*) under three management and exercise contexts: show jumping competition, race training and racing, and leisure riding across the riding season. The study used a paired pre/post sampling design within each horse. For show jumping horses, samples were collected immediately before and shortly after a competition round. For racehorses, samples were collected at three periods of the season (start of the season, mid-season after several weeks of structured training, and around official races), with paired pre/post sampling for each training or race event. For leisure horses, samples were collected at rest before the start and after the end of the riding season. Sample size was determined by animal availability within each cohort; no apriori power calculation was performed. No animals were euthanised.

### Animals and husbandry

The study included show jumping horses (*n* = 17), racehorses (*n* = 43 enrolled), and recreational leisure horses (*n* = 14). Racehorses were managed under a single training program at one racing yard, and leisure horses were housed in a single stable. Show jumping horses were sourced from multiple stables and managed according to each facility’s routine care, diet, and training protocols. Baseline animal characteristics and cohort composition are summarized in Supplementary Table 1. Horses were sampled only when they were in regular work and considered clinically fit for the scheduled activity during routine veterinary oversight; horses with overt acute illness or injury requiring exercise restriction were not intentionally sampled.

The show jumping cohort comprised 17 horses (9 mares and 8 geldings) aged 4–15 years (median 6 years). Breeds included Dutch Warmblood (KWPN; *n* = 11), Holsteiner (*n* = 1), Belgian Warmblood (*n* = 1), Polish Warmblood (*n* = 1), Anglo European Studbook (AES; *n* = 1), Małopolski (*n* = 1), and one horse of unknown breed (*n* = 1). Horses competed in P-class show jumping (courses up to 110 cm) and followed their usual stable-specific training and feeding routines.

The racehorse cohort comprised Thoroughbreds (*n* = 12) and Arabians (*n* = 31) (*n* = 43; 14 mares and 29 stallions), aged 2–7 years (median 3 years). Horses were stabled individually on bedding and were turned out daily when weather permitted. Nutrition was standardized within the yard and consisted of hay, commercial concentrates, and vitamin–mineral supplementation, with ad libitum access to fresh water. Training was performed under saddle six days per week when weather conditions were suitable. Two horses did not contribute complete paired observations for any biomarker and were not included in inferential analyses; the primary analytic dataset therefore comprised 41 racehorses (as reflected in the Results).

The leisure cohort comprised 14 horses (9 mares and 5 geldings) aged 9–27 years (median 12.5 years), used for regular leisure riding. Breeds included Polish Warmblood (*n* = 3), Małopolski (*n* = 4), Fjord (*n* = 3), Arabian (*n* = 2), Felin Pony (*n* = 1), and one horse of unknown breed (*n* = 1). Horses were housed in individual stalls with regular access to paddocks outside training sessions and were maintained on a uniform diet within the stable.

### Exercise and training protocols

Show jumping horses were trained and competed according to individual schedules determined by their riders and stables, with routine flatwork and jumping practice. All horses were sampled on a competition day during participation in a P-class (≤ 110 cm) round.

Racehorse training followed a structured program. A typical session consisted of a warm-up phase (10 min walking under saddle, followed by 800 m trotting and 800 m slow canter), a conditioning phase (1,400-2,400 m canter at a moderate pace of 16–22 s per 200 m), and a high-intensity phase comprising full-gallop work over 500-1,200 m at 80%–100% of maximal speed (13–15 s per 200 m), followed by cool-down walking for 30–40 min on a mechanical walker. High-intensity gallop sessions were incorporated approximately every 5–10 days in accordance with race conditioning practice.

Leisure horses underwent moderate-intensity riding sessions six times per week. Sessions consisted predominantly of flatwork and endurance-oriented riding, with basic jumping incorporated depending on rider level and routine stable practice.

### Blood collection and timing

Venous blood was collected from the jugular vein using sterile single-use needles (BD Vacutainer Precisionglide, 20 G [0.9 × 38 mm]) into vacuum tubes (BD Vacutainer, Franklin Lakes, NJ, USA). EDTA-coated tubes were collected for routine hematological monitoring, and serum tubes containing a clot activator were collected for biomarker analysis. Blood sampling was performed by a licensed veterinarian under calm, standardized handling conditions.

Sampling time points were selected to capture resting baseline conditions and short-term post-exercise responses while minimizing confounding by recent exercise and diurnal variability. For show jumping horses, blood was collected approximately 60 min before the competition round (at rest in the stable) and 30 min after completion of the course, following cool-down and after heart rate had stabilized. Competitions were held between 10:00 and 14:00, and horses were not exercised earlier on the same day before the competition.

For racehorses, paired sampling was performed at three periods: (i) T1, at the start of the training season; (ii) T2, after 6–10 weeks of regular high-intensity training; and (iii) R, before and after official races. Pre-exercise samples were collected at rest in the stable between 06:00 and 08:00, at least 24 h after the last training session. Post-exercise samples were collected 30–40 min after the end of training or racing, after cool-down and physiological recovery.

For leisure horses, blood was sampled twice: in June (before the start of the riding season) and in September (after the end of the riding season). On both occasions, horses were sampled at rest between 08:00 and 10:00, at least 24 h after the last exercise session and before feeding to reduce acute metabolic variability.

Serum tubes were processed within 5 h of collection. Samples were centrifuged at 3,000 × g for 10 min at 4 °C, and serum was separated and stored at − 80 °C until analysis.

### Biomarker quantification by ELISA

Serum concentrations of serotonin (5-hydroxytryptamine; 5-HT), brain-derived neurotrophic factor (BDNF), cortisol, and oxytocin were determined using commercially available equine enzyme-linked immunosorbent assay (ELISA) kits (ELK Biotechnology, Denver, USA) according to the manufacturer’s instructions. Standard curves were generated from kit-provided calibrators for each plate. Optical density was measured at 450 nm using a microplate reader (Multiscan Reader, Labsystem, Helsinki, Finland) with Genesis V software (v3.00). Manufacturer-reported analytical performance characteristics for equine serum were as follows.

For serotonin (ELK0384), sensitivity was 5.85 ng/mL; intra-assay precision ≤ 8%; inter-assay precision ≤ 10%; recovery 93%–107% (mean 100%); and linearity under serial dilution: 1:2 (95%–106%), 1:4 (87%–101%), 1:8 (82%–97%), and 1:16 (87%–102%).

For BDNF (ELK10132), sensitivity was 0.058 ng/mL; intra-assay precision ≤ 8%; inter-assay precision ≤ 10%; recovery 83%–98% (mean 91%); and linearity: 1:2 (89%–97%), 1:4 (86%–102%), 1:8 (94%–106%), and 1:16 (85%–97%).

For cortisol (ELK8554), sensitivity was 0.95 ng/mL; intra-assay precision ≤ 8%; inter-assay precision ≤ 10%; recovery 85%–97% (mean 91%); and linearity: 1:2 (85%–92%), 1:4 (79%–96%), 1:8 (89%–99%), and 1:16 (85%–95%).

For oxytocin (ELK11231), sensitivity was 5.53 pg/mL; intra-assay precision ≤ 8%; inter-assay precision ≤ 10%; recovery 87–99% (mean 93%); and linearity: 1:2 (88%–104%), 1:4 (84%–98%), 1:8 (95%–105%), and 1:16 (88%–102%).

### Data Analysis

All analyses were performed in Python (pandas v2.3, NumPy v2.4, SciPy v1.16, and statsmodels v0.14). For each horse and marker, the primary outcome was the post-to-pre ratio $\:r={y}_{\mathrm{post}}/{y}_{\mathrm{pre}}$. Because ratios are typically right-skewed and multiplicative, inferential analyses were conducted on the natural-log scale as $\:{\Delta\:}=\mathrm{l}\mathrm{n}\left(r\right)=\mathrm{l}\mathrm{n}\left({y}_{\mathrm{post}}\right)-\mathrm{l}\mathrm{n}\left({y}_{\mathrm{pre}}\right)$. Model estimates were back-transformed and reported as geometric mean post/pre ratios with 95% confidence intervals (95% CI) by exponentiating the estimated mean and its confidence limits on the log scale; a ratio of 1.0 indicates no change. Percent change was calculated as $\:(\mathrm{ratio}-1)\times\:100$. Records with $\:{y}_{\mathrm{pre}}\le\:0\:$or $\:{y}_{\mathrm{post}}\le\:0$ were excluded for the affected marker. For leisure horses, this ratio was used only as a scale-free summary of paired resting change across the riding season and was not interpreted as an acute exercise response.

Baseline characteristics (numbers of horses, sex distribution, age, and breed) and data completeness (paired pre/post availability per marker) were summarized descriptively and reported in Supplementary Tables 1–2.

For show jumping and leisure horses, each horse contributed one paired observation. Primary within-discipline inference for each marker used an intercept-only model on $\:{\Delta\:}$: $\:{{\Delta\:}}_{i}=\mu\:+{\epsilon\:}_{i}$, with $\:{\epsilon\:}_{i}\sim\:\mathcal{N}(0,{\sigma\:}^{2})$, testing $\:{H}_{0}:\mu\:=0$ (equivalently, post/pre $\:=1$). Sex-stratified intercept-only models were fitted as supplementary analyses within each sex subgroup. For show jumping horses, breed-adjusted analyses were additionally performed for each marker using ordinary least squares (OLS) with categorical fixed effects for sex and breed: $\:{{\Delta\:}}_{i}={\beta\:}_{0}+{\beta\:}_{s\left(i\right)}+{\beta\:}_{b\left(i\right)}+{\epsilon\:}_{i}$. Sex-specific marginal means were obtained by fixing sex to a given level while standardizing predictions over the observed breed distribution in the analytic dataset, and then back-transforming to the post/pre ratio scale. Low-frequency breed categories were pooled into “Other” according to a prespecified minimum count threshold to improve model stability.

For racehorses, repeated paired observations were available across sessions (T1, T2, race day). Primary racehorse inference for each marker used a linear mixed-effects model with a random intercept for horse: $\:{{\Delta\:}}_{ij}={\beta\:}_{0}+{\beta\:}_{c\left(j\right)}+{\beta\:}_{s\left(i\right)}+{\beta\:}_{b\left(i\right)}+{u}_{i}+{\epsilon\:}_{ij}$, where $\:c\left(j\right)$denotes session, $\:s\left(i\right)$denotes sex, $\:b\left(i\right)$ denotes breed, $\:{u}_{i}\sim\:\mathcal{N}(0,{\tau\:}^{2})$, and $\:{\epsilon\:}_{ij}\sim\:\mathcal{N}(0,{\sigma\:}^{2})$. Fixed-effect marginal means were computed for each session-by-sex combination by setting session and sex to the target levels while standardizing over the observed breed distribution; results were back-transformed to post/pre ratios with 95% CI derived from the fixed-effect variance–covariance matrix.

Supplementary racehorse analyses were conducted using intercept-only OLS models within selected subgroups (session × sex for serotonin and sex-stratified pooled analyses across sessions for other markers); these supplementary models did not account for within-horse clustering.

**Fig. 1:**
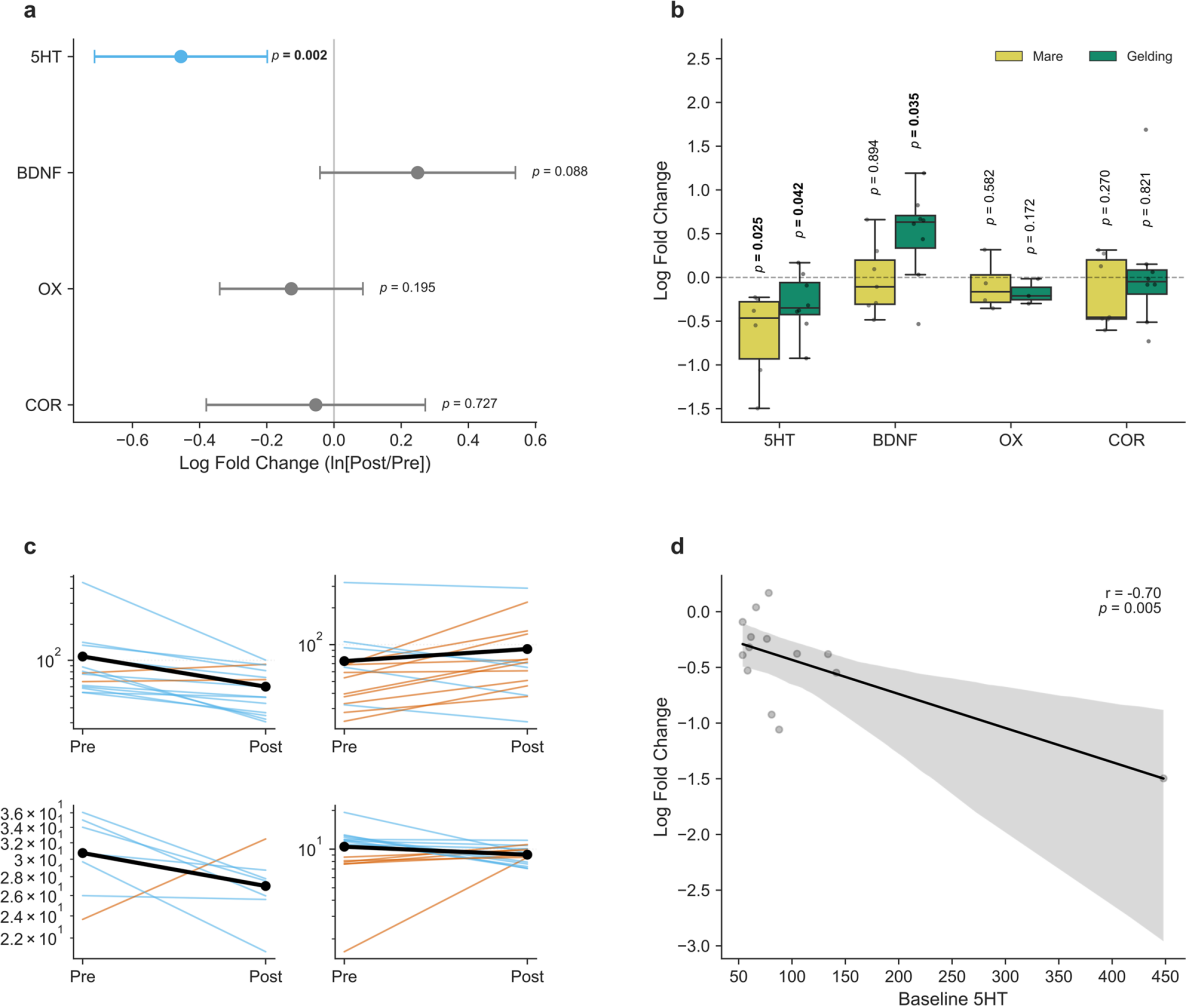
Serum biomarker responses to show jumping competition. **a** Overall response expressed as log fold change $\:{\Delta\:}=\mathrm{l}\mathrm{n}(\mathrm{p}\mathrm{o}\mathrm{s}\mathrm{t}/\mathrm{p}\mathrm{r}\mathrm{e})$for serotonin (5-HT), brain-derived neurotrophic factor (BDNF), oxytocin (OX), and cortisol (COR). Points denote mean $\:{\Delta\:}$ and horizontal bars denote 95% confidence intervals calculated using the *t* distribution. **b** Distribution of $\:{\Delta\:}$ by sex. Boxes indicate median and interquartile range, whiskers indicate 1.5 × interquartile range, and points represent individual horses. *P* values above boxes correspond to within-sex tests of $\:{\Delta\:}$ against 0 derived from intercept-only models (Supplementary Table 5). **c** Paired serum concentrations at pre and post sampling for each biomarker on a log-scaled y-axis. Lines represent individual horses, vermilion indicates increases and sky blue indicates decreases, and the black line indicates the group mean. **d** Baseline dependency for 5-HT shown as baseline (pre) concentration versus $\:{\Delta\:}$. The line shows ordinary least squares regression with 95% confidence band, and Pearson correlation coefficient and two-sided *p* value are shown in the panel

**Fig. 2:**
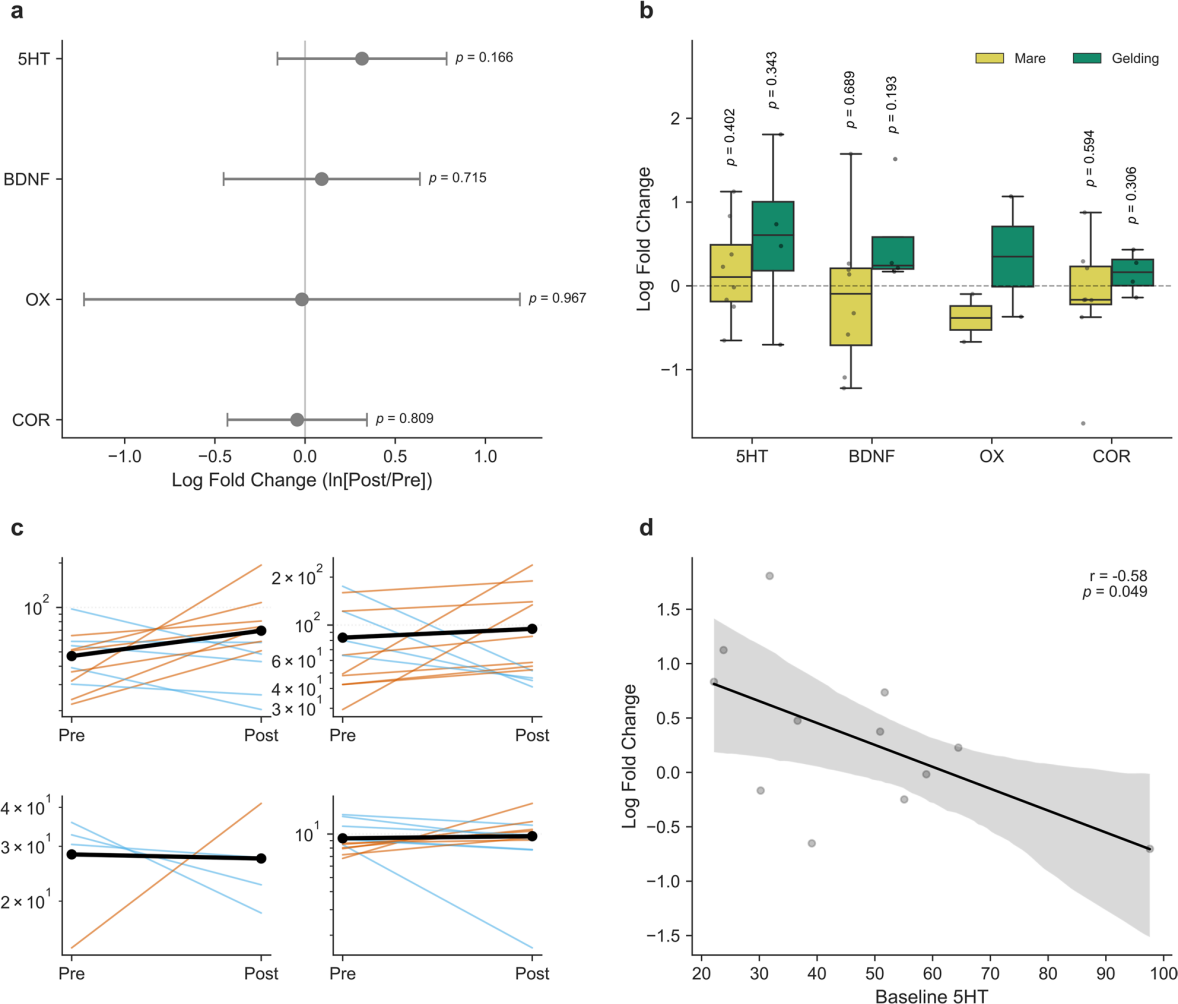
Serum biomarker responses across the leisure riding season. **a** Overall response expressed as log fold change $\:{\Delta\:}=\mathrm{l}\mathrm{n}(\mathrm{p}\mathrm{o}\mathrm{s}\mathrm{t}/\mathrm{p}\mathrm{r}\mathrm{e})$for 5-HT, BDNF, OX, and COR. Points denote mean $\:{\Delta\:}$ and horizontal bars denote 95% confidence intervals calculated using the *t* distribution. **b** Distribution of $\:{\Delta\:}$ by sex. Boxes indicate median and interquartile range, whiskers indicate 1.5 × interquartile range, and points represent individual horses. *P* values above boxes correspond to within-sex tests of $\:{\Delta\:}$ against 0 derived from intercept-only models (Supplementary Table 7). **c** Paired serum concentrations at pre and post sampling for each biomarker on a log-scaled y-axis. Lines represent individual horses, vermilion indicates increases and sky blue indicates decreases, and the black line indicates the group mean. **d** Baseline dependency for 5-HT shown as baseline (pre) concentration versus $\:{\Delta\:}$. The line shows ordinary least squares regression with 95% confidence band, and Pearson correlation coefficient and two-sided *p* value are shown in the panel

**Fig. 3:**
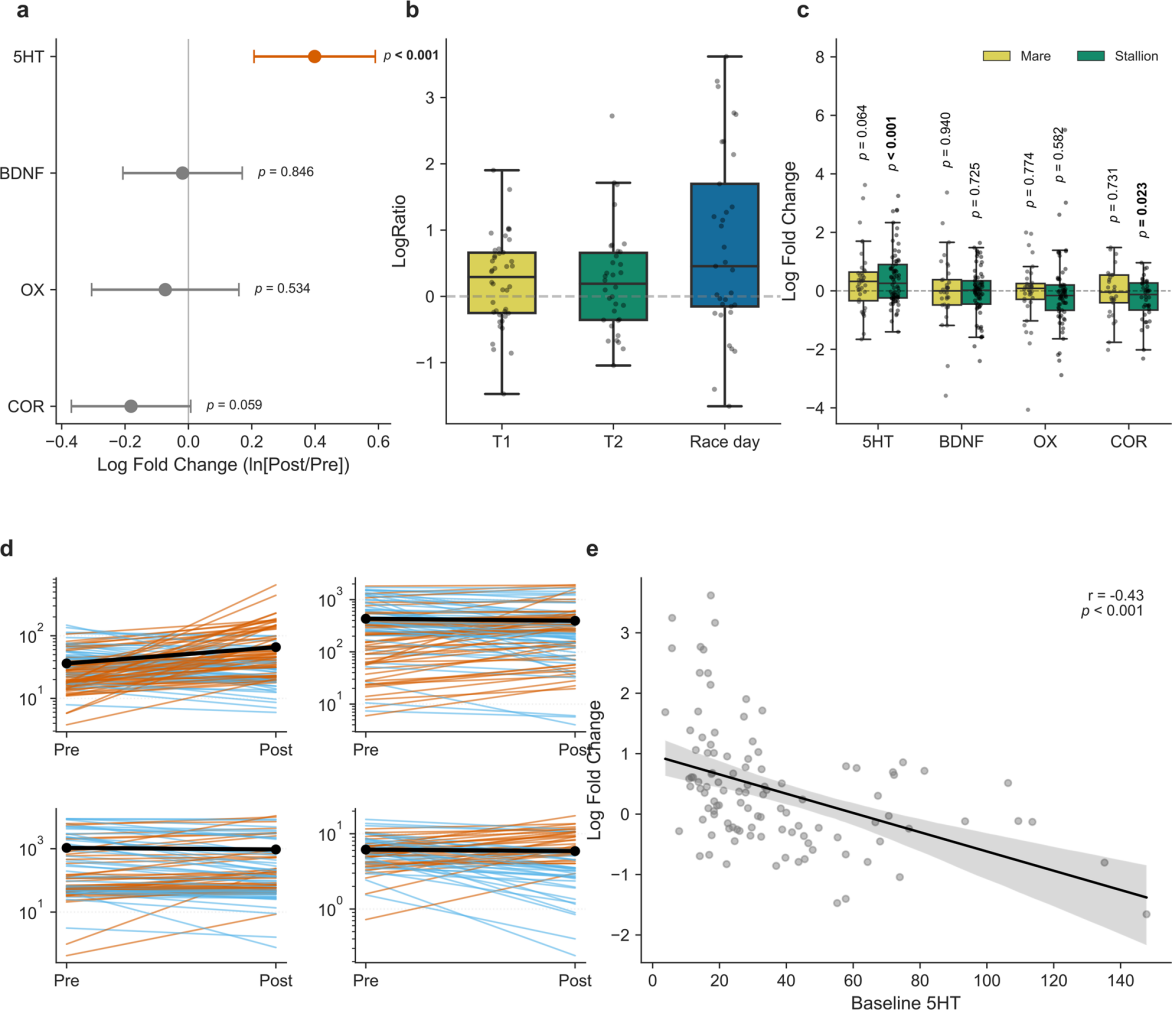
Serum biomarker responses in racehorses across the training season and on race day. **a** Descriptive pooled overall response expressed as log fold change $\:{\Delta\:}=\mathrm{l}\mathrm{n}(\mathrm{p}\mathrm{o}\mathrm{s}\mathrm{t}/\mathrm{p}\mathrm{r}\mathrm{e})\:$for 5-HT, BDNF, OX, and COR across all sessions. Points denote mean $\:{\Delta\:}$ and horizontal bars denote 95% confidence intervals calculated using the *t* distribution. **b** Distribution of 5-HT $\:{\Delta\:}$ by training session (T1, T2, and race day). Boxes indicate median and interquartile range, whiskers indicate 1.5 × interquartile range, and points represent individual paired sessions. **c** Distribution of $\:{\Delta\:}$ by sex for each biomarker. Boxes indicate median and interquartile range, whiskers indicate 1.5 × interquartile range, and points represent individual paired sessions. *P* values above boxes correspond to within-sex pooled OLS tests of $\:{\Delta\:}$ against 0 across sessions (Supplementary Table 9) and do not account for within-horse clustering. **d** Paired serum concentrations at pre and post sampling for each biomarker on a log-scaled y-axis across all sessions. Lines represent individual paired sessions, vermilion indicates increases and sky blue indicates decreases, and the black line indicates the mean trajectory. **e** Baseline dependency for 5-HT shown as baseline (pre) concentration versus $\:{\Delta\:}\:$across all sessions. The line shows ordinary least squares regression with 95% confidence band, and Pearson correlation coefficient and two-sided *p* value are shown in the panel

Visual summaries were generated for each discipline to support interpretation. Forest plots (Figs. 1a, 2a and 3a) display mean $\:{\Delta\:}$ with 95% CI computed using the $\:t$-distribution and two-sided one-sample $\:t$-tests versus 0; for show jumping and leisure these tests are numerically equivalent to the intercept-only models, whereas for racehorses Fig. 3a is a descriptive pooled summary across sessions and does not account for within-horse clustering. Box-and-whisker plots with overlaid individual points (Figs. 1b, 2b and 3c) display the distribution of $\:{\Delta\:}$ by sex for each marker, with a dashed reference line at 0. Paired pre/post “spaghetti” plots (Figs. 1c, 2c and 3d) display individual trajectories of raw concentrations on a log-scaled y-axis (increase vs. decrease indicated by line color), with an overlaid mean trajectory. Baseline dependency plots (Figs. 1d, 2d and 3e) assess the relationship between baseline 5-HT (pre) and $\:{\Delta\:}$using Pearson correlation and an ordinary least squares regression line with 95% CI; the corresponding correlation coefficient ($\:r$) and two-sided *p* value are displayed in each panel. For racehorses, an additional distribution plot of 5-HT $\:{\Delta\:}$ by session (Fig. 3b) was used to visualize session-specific differences.

All hypothesis tests were two-sided with $\:\alpha\:=0.05$. Analyses used complete cases for each marker and model (no imputation); sample sizes therefore varied across markers owing to missing pre or post measurements and, where applicable, missing covariate data.

## Results

Baseline characteristics by discipline are reported in Supplementary Tables 1, and paired pre/post data completeness by marker is reported in Supplementary Table 2. Show jumping included 17 horses (9 mares, 8 geldings), leisure included 14 horses (9 mares, 5 geldings), and race included 41 horses (14 mares, 27 stallions). Oxytocin had the lowest paired availability in show jumping and leisure, and cortisol had substantial missingness in race sessions (Supplementary Table 2).

### Show jumping horses

Pooled within-discipline post/pre ratios for show jumping are summarized in Supplementary Tables 3 and visualized on the $\:{\Delta\:}$scale in Fig. 1a. Serotonin (5-hydroxytryptamine, 5-HT) decreased after competition (ratio 0.63, 95% CI 0.49–0.82; *p* = 0.002; Supplementary Table 3; Fig. 1a). In the pooled analysis, BDNF showed a non-significant increase (1.28, 0.96–1.72; *p* = 0.088), whereas cortisol (0.95, 0.68–1.31; *p* = 0.727) and oxytocin (0.88, 0.71–1.09; *p* = 0.195) did not differ from unity (Supplementary Table 3; Fig. 1a). Sex-stratified post/pre ratios are provided in Supplementary Tables 5 and are consistent with the sex-disaggregated distributions in Fig. 1b. The 5-HT decrease was observed in mares (0.52, 0.30–0.88; *p* = 0.02) and geldings (0.74, 0.55–0.99; *p* = 0.042) (Supplementary Table 5; Fig. 1b). BDNF increased in geldings (1.63, 1.05–2.52; *p* = 0.035) but not in mares (0.98, 0.68–1.42; *p* = 0.894) (Supplementary Table 5; Fig. 1b). Individual paired trajectories on a log-scaled axis (Fig. 1c) showed heterogeneous responses across horses, with the mean trajectory indicating a net decrease for 5-HT and a net increase for BDNF. Baseline dependency analysis indicated that higher baseline 5-HT was associated with a more negative $\:{\Delta\:}$ (Pearson $\:r=-0.70$, *p* = 0.005; Fig. 1d).

Breed-adjusted, sex-specific post/pre ratios (standardized over the observed breed distribution) are reported in Supplementary Table 6. After adjustment, the 5-HT decrease remained significant in mares (0.47, 0.25–0.88; *p* = 0.026) but was not significant in geldings (0.79, 0.47–1.34; *p* = 0.321), whereas the BDNF increase persisted in geldings (1.73, 1.12–2.67; *p* = 0.021) with no evidence of change in mares (0.91, 0.57–1.47; *p* = 0.665) (Supplementary Table 6).

### Leisure horses

Pooled within-discipline post/pre ratios for leisure are summarized in Supplementary Tables 3 and visualized in Fig. 2a. No marker differed significantly from unity in the pooled analyses (5-HT: 1.37, 0.86–2.19; *p* = 0.166; BDNF: 1.10, 0.64–1.89; *p* = 0.715; cortisol: 0.96, 0.65–1.41; *p* = 0.809; oxytocin: 0.98, 0.29–3.29; *p* = 0.967; Supplementary Table 3; Fig. 2a). Sex-stratified results for leisure are provided in Supplementary Tables 7 and are consistent with the distributions in Fig. 2b. No statistically significant within-sex changes were detected for 5-HT, BDNF, or cortisol (all *p* ≥ 0.193), and sex-stratified oxytocin estimates were not available because $\:n<3$within each sex (Supplementary Table 7). Paired trajectories (Fig. 2c) did not show a consistent directional shift across markers, and the baseline dependency plot indicated a negative association between baseline 5-HT and $\:{\Delta\:}\:$(Pearson $\:r=-0.58$, *p* = 0.049; Fig. 2d).

### Racehorses

Primary racehorse inference was based on mixed-effects models; session-by-sex marginal mean post/pre ratios are reported in Supplementary Table 4. Serotonin increased on race day in both mares (1.95, 1.26–3.02; *p* = 0.003) and stallions (2.21, 1.53–3.18; *p* < 0.001), whereas 5-HT did not differ from unity after T1 or T2 in either sex (all *p* ≥ 0.112; Supplementary Table 4). The session-specific distributions of 5-HT $\:{\Delta\:}$ (Fig. 3b) were concordant with this pattern, showing larger positive shifts on race day compared with T1 and T2. The pooled descriptive forest plot across sessions (Fig. 3a) also showed a positive overall mean $\:{\Delta\:}$ for 5-HT; however, this pooled visualization does not account for within-horse clustering and was not used for primary inference.

For BDNF and oxytocin, mixed-model marginal means remained close to unity across sessions and sexes (BDNF: 0.89–1.11; oxytocin: 0.87–0.97; all *p* ≥ 0.507; Supplementary Table 4), consistent with the near-zero centered distributions in Fig. 3c and the mixed directionality of individual paired trajectories in Fig. 3d. For cortisol, stallions showed lower post/pre ratios after T2 (0.70, 0.49–1.01; *p* = 0.059) and on race day (0.71, 0.50–1.02; *p* = 0.064), whereas mares showed no significant session-specific changes (all *p* ≥ 0.482; Supplementary Table 4); these patterns correspond to the slight left shift for stallion cortisol in Fig. 3c and the predominance of decreasing individual trajectories in a subset of stallion sessions in Fig. 3d. Baseline dependency analysis across race sessions indicated that higher baseline 5-HT was associated with a smaller increase (more negative $\:{\Delta\:}$) (Pearson $\:r=-0.43$, *p* < 0.001; Fig. 3e). Supplementary OLS analyses that did not model within-horse clustering are provided for transparency (Supplementary Tables 8–9). In session-by-sex OLS analyses for 5-HT (Supplementary Table 8), stallions showed increases after T1 (*p* = 0.004) and on race day (*p* = 0.023), whereas mares showed a non-significant race-day increase (*p* = 0.077). In pooled OLS analyses across sessions within sex (Supplementary Table 9), stallions showed increased 5-HT (*p* < 0.001) and decreased cortisol (*p* = 0.023), whereas mares did not show significant pooled changes (Supplementary Table 9).

## Discussion

Across the studied contexts, paired biomarker profiles revealed discipline-specific stress-related endocrine and neuromodulatory responses, but these responses varied markedly among individual horses. Serotonin provided the most consistent signal, with a post-competition decrease in show jumping and a marked increase on race day in racehorses when repeated observations were modeled with mixed effects. Leisure horses sampled at rest before and after the riding season did not show significant changes in any marker. Worth, mentioning is that cortisol exhibits a pronounced circadian rhythm with a morning peak and decline throughout the day, along with moderate seasonality (typically higher in winter/early spring) [6, 17, 19, 22, 23], while BDNF [21, 34] and serotonin [20, 28, 31] turnover also show diurnal and photoperiod-related variation; in contrast, oxytocin patterns are inconsistent and limited by methodological constraints [28, 31]. Thus, these temporal and seasonal factors should be taken into account when interpreting the results.

### Serotonergic responses across disciplines and baseline dependency

Serotonin showed opposite directions of change between show jumping and race day. Show jumping was associated with decreased serum 5-HT after competition in pooled and sex-stratified models. Breed-adjusted models retained a significant decrease in mares and attenuated the gelding estimate, indicating that sex-specific inference in a small cohort was sensitive to covariate structure and limited precision. Show jumping competition combines high technical demands with environmental novelty and can incorporate an emotional component that modulates endocrine and monoaminergic responses [36]. Arfuso et al. reported competition-related shifts in peripheral modulators of serotonergic function and neurohumoral factors in jumper horses and interpreted the pattern as indirect support for serotonergic involvement in fatigue under competitive conditions [37]. In contrast to the present post-competition decrease, obstacle-course studies in athletic horses reported increases in peripheral serotonergic markers immediately after exercise and persistent elevation at 30 min, emphasizing that direction and magnitude of peripheral 5-HT change depend on sampling window and biological matrix [38]. Acute handling stressors can also influence serotonergic measures, as short as transport followed by slaughter reduced circulating 5-HT while increasing cortisol and serotonin receptor expression in peripheral blood cells [39]. The inverse association between baseline 5-HT and ln(post/pre) in show jumping is compatible with baseline-dependent responsiveness, including constrained dynamic range and regression-to-the-mean effects.

Racehorses showed a pronounced 5-HT increase on race day in mares and stallions in mixed-effects models, whereas no significant deviations from unity were detected after T1 or T2 in either sex. Training and simulated race protocols have been reported to induce transient increases in circulating 5-HT with recovery toward baseline within hours, supporting context sensitivity of peripheral serotonergic responses during conditioning [40]. Coordinated hormonal coping responses during exercise stress provide a framework in which serotonergic changes reflect one component of an integrated adaptation profile shaped by exercise characteristics and emotional load [36]. Sex-stratified show jumping models indicated 5-HT decreases in both mares and geldings, and breed-adjusted models supported a more stable decrease in mares. Survey-based data report sex-related differences in some non-ridden behaviors and handling contexts, whereas sexually dimorphic ridden behavior has not been consistently supported, suggesting caution when linking sex to competition-related neuroendocrine responses [41]. Gonadal steroids modulate serotonergic physiology and stress-axis responsivity in experimental models, supporting biological plausibility for estradiol- and progesterone-related modulation of serotonergic dynamics in mares, although estrous-cycle staging was not available for formal testing in present study [42, 43].

Our findings support the hypothesis that serotonergic responses differ by equestrian discipline, with the direction and magnitude of 5-HT change reflecting the competition context rather than exercise alone.

### BDNF patterns and exercise modality

BDNF did not show significant post/pre changes across race sessions in mixed-effects models, and leisure horses did not show significant change (Supplementary Tables 3, 4, and 7). High-intensity exercise can produce variable peripheral BDNF responses, and human data indicate that serum BDNF may remain unchanged after training interventions and may decrease after Wingate-type anaerobic testing, highlighting sensitivity to exercise test characteristics and recovery interval [44]. Evidence from experimental and clinical literature links sustained stress exposure and glucocorticoid signaling with reduced BDNF in stress-related phenotypes, including decreased hippocampal BDNF expression after chronic social stress paradigms and lower peripheral BDNF in anxiety-associated states [34, 35, 45, 46]. The absence of a detectable BDNF response in race sessions is compatible with several non-exclusive explanations, including exercise-modality effects, baseline variability, and sampling time relative to peak BDNF kinetics.

Show jumping showed a different pattern. Sex-stratified analyses indicated a BDNF increase in geldings, and breed-adjusted models retained the gelding-associated increase with no evidence of change in mares. Basal serum BDNF has been reported to be higher in active polo horses than in sedentary horses, and absence of differences across training-intensity periods has been attributed in part to delayed sampling after exercise completion, supporting a role for sampling window when interpreting peripheral BDNF dynamics [47]. The lack of a BDNF signal in leisure horses may reflect lower workload intensity and a seasonal resting sampling design that targets longer-term adaptation rather than acute post-exercise kinetics. The gelding-associated BDNF increase in show jumping supports a hypothesis of discipline-specific modulation of peripheral BDNF, while mechanistic attribution to neuroplasticity-related processes requires replication in larger cohorts and integration with behavioral or performance measures.

### Oxytocin and cortisol in relation to arousal, social context, and sex

Oxytocin did not show significant changes in the primary models across cohorts, and inference in show jumping and leisure horses was limited by missing paired availability. Oxytocin is implicated in social bonding and stress buffering, and oxytocinergic signaling is sensitive to arousal state and social context [48, 49]. Interpretation of circulating oxytocin requires caution because central release can occur without measurable changes in blood, and assay performance and pre-analytical handling can influence detectability [49]. The absence of detectable oxytocin change under the present sampling scheme is compatible with a temporal mismatch relative to rapid oxytocin kinetics and with substantial biological heterogeneity. A pilot field study of equine-assisted activities similarly reported no significant changes in plasma oxytocin across repeated time points around sessions and discussed short oxytocin half-life and sampling timing as potential contributors [50]. Evidence from temperament studies suggests that baseline plasma oxytocin and serotonin can relate to trainability and social reactivity in horses, supporting a role for stable between-horse differences even when acute within-horse changes are small [28]. Importantly, peripheral oxytocin quantification remains highly method-dependent: immunoassays may differ in specificity and can capture oxytocin-related immunoreactive products, and reported concentrations may vary substantially depending on sample preparation (e.g., extraction/clean-up) and platform. Accordingly, we interpret serum oxytocin primarily as a within-study marker and avoid strong cross-study comparisons unless analytical workflows and validation are comparable [51].

Cortisol ratios did not differ from unity in show jumping or leisure horses, and racehorse mixed-effects estimates suggested lower cortisol ratios in stallions during T2 and race day without meeting the prespecified significance threshold. Studies in Arabian race and endurance horses have reported cortisol increases after training and larger increases after competition, supporting sensitivity of cortisol to exercise intensity and context under standardized pre and post sampling [52]. Competition studies measuring salivary cortisol also report transient post-competition increases with return toward baseline within approximately 1 h, supporting the importance of sampling window and biological matrix when interpreting serum cortisol after cool-down [25]. Interpretation of circulating serotonin and cortisol requires caution because peripheral serotonin constitutes the dominant body pool and regulates multiple organ systems outside the central nervous system, and glucocorticoid readouts vary across stimuli and sample types [21, 30]. Endocrine responses to workload can differ across axes, as intense leisure exploitation in horses was associated with higher testosterone and an increased testosterone to cortisol ratio while cortisol remained unchanged, indicating context-dependent coupling of HPG and HPA markers [53]. Sex steroid effects on serotonergic regulation are biologically plausible, including evidence that high-dose testosterone treatment alters serotonin transporter binding in humans, although direct translation to equine peripheral serotonin dynamics is uncertain [54]. Oxytocin effects can also depend on endocrine context in humans, as oxytocin moderated associations between testosterone to cortisol ratio and trust-related behavior in a controlled experimental paradigm [55]. Direct measurement of sex steroids was not performed in the present cohort, so sex-hormone mechanisms remain hypotheses rather than demonstrated pathways.

### Integrated interpretation and contextual relevance

The combined biomarker profile supports an interpretive framework in which peripheral serotonergic responsiveness distinguishes competition contexts more clearly than cortisol or oxytocin under the selected sampling schedule. Show jumping competition was associated with decreased 5-HT and a gelding-associated increase in BDNF, whereas race day was associated with a marked 5-HT increase in mares and stallions in mixed-effects models. Negative baseline dependency of ln(post/pre) for 5-HT across cohorts indicates that baseline concentration influenced the magnitude of paired change, which supports baseline-aware interpretation and is also compatible with constrained dynamic range and regression-to-the-mean effects. Multi-marker evaluation may therefore help characterize discipline-specific patterns and between-horse variability that are not reflected by a single endocrine readout, but the present data do not by themselves establish welfare status. The interpretation of circulating 5-HT benefits from explicit recognition of the expanded peripheral serotonin biology, because most serotonin is located outside the central nervous system and regulates multiple peripheral organ systems [30].

### Limitations and future directions

Modest sample sizes in show jumping and leisure horses reduced precision, oxytocin had substantial missingness in both cohorts, and racehorse cortisol availability was lower than for other markers. Circulating biomarkers integrate multiple physiological sources and provide indirect information about central affective processing, and circulating 5-HT in particular reflects a predominantly peripheral pool with broad systemic functions rather than a direct central readout. Endocrine interpretation is additionally shaped by stimulus characteristics and sample matrix, and glucocorticoid behavior can differ across stressors and measurement contexts, supporting cautious inference from a single sampling window. The observational design incorporated real-world management, and residual confounding by temperament, transport exposure, estrous-cycle stage, and feeding schedule cannot be excluded. Breed adjustment altered selected subgroup inferences in show jumping, supporting cautious interpretation of sex-specific patterns in small cohorts. Replication in larger cohorts with standardized documentation of transport exposure, estrous-cycle staging, and expanded post-exercise sampling windows would strengthen mechanistic inference and improve alignment between biomarker kinetics and sampling time. Additionally, the integration of endocrine markers with continuous or field-friendly welfare monitoring tools (e.g., wearable sensor platforms) is a promising direction to improve temporal resolution and interpretability under real training conditions [56]. Potential circadian and seasonal variation, particularly for cortisol, also cannot be fully disentangled from discipline effects in the present design, although sampling within each cohort was restricted to relatively narrow time windows.

Because circulating BDNF is strongly platelet-associated and is released during coagulation, serum BDNF concentrations can be sensitive to pre-analytical conditions, particularly clotting duration and temperature, as well as the time between collection and centrifugation. Although all samples in this study were handled using one protocol, residual variation in these factors may add analytical noise and bias small exercise-related effects toward the null. Future studies should consider tighter standardization and reporting of clotting time and, where feasible, complementary matrices (e.g., plasma) to better characterise the relevant BDNF pool [57]. Similarly, peripheral serotonin is predominantly platelet-associated, and measured concentrations can be strongly influenced by platelet activation and by mechanical/thermal stress and processing delays. Differences in pre-analytical procedures are a major source of between-study variability [58]. This platelet dependence should be considered when interpreting serum 5-HT as a welfare-related marker, particularly when expected effects are small.

Future work may benefit from reporting tighter pre-analytical control (time-to-processing, centrifugation strategy) and/or adding platelet-poor plasma approaches to better isolate the free circulating fraction.

## Conclusions

Serotonin showed the clearest discipline-dependent responsiveness, with a decrease after show jumping competition and a marked increase on race day in mixed-effects models. BDNF increases were restricted to show jumping geldings in sex-stratified and breed-adjusted analyses, whereas race and leisure cohorts showed no significant BDNF change. Oxytocin post to pre ratios remained close to a post/pre ratio of 1.0 across cohorts under the selected sampling schedule, although limited paired availability in show jumping and leisure horses reduced precision. Cortisol ratios were stable in show jumping and leisure horses, and racehorse mixed-effects models suggested lower stallion ratios during T2 and race day without meeting the prespecified significance threshold. Supplementary OLS analyses that did not account for within-horse clustering indicated a stallion-associated cortisol decrease when race sessions were pooled, supporting cautious interpretation of cortisol findings across model structures. Overall, these findings support cautious use of multi-marker profiling to characterize discipline-dependent neuroendocrine dynamics and baseline-related variation under the applied sampling framework, rather than direct inference about welfare status.

## Supplementary Information

Supplementary Material 1.

Supplementary Material 2.

Supplementary Material 3.

Supplementary Material 4.

## Data Availability

Datasets are deposited at https://repod.icm.edu.pl/dataset.xhtml?persistentId=doi:10.18150/IEXY65 RepOD repository.

## Abbreviations

5-HT: 5-hydroxytryptamine (serotonin)
AAM: Author Accepted Manuscript
AES: Anglo European Studbook
ARRIVE: Animal Research: Reporting of In Vivo Experiments
BDNF: Brain-derived neurotrophic factor
CC BY: Creative Commons Attribution license
CI: Confidence interval
COR: Cortisol
EDTA: Ethylenediaminetetraacetic acid
ELISA: Enzyme-linked immunosorbent assay
HPA: Hypothalamic-pituitary-adrenal
HPG: Hypothalamic-pituitary-gonadal
HR: Heart rate
HRV: Heart rate variability
IRT: Infrared thermography
KWPN: Koninklijk Warmbloed Paardenstamboek Nederland (Dutch Warmblood)
ln: Natural logarithm
OLS: Ordinary least squares
OPUS 21: National Science Centre Poland grant program OPUS 21
OX: Oxytocin
R: Race day
SGGW: Warsaw University of Life Sciences (Szkoła Główna Gospodarstwa Wiejskiego)
T1: Start of the training season
T2: After 6–8 weeks of high-intensity training
Δ: ln(post/pre) change
