# Supplementary material for: Beyond cortisol: evaluating serotonin, brain-derived neurotrophic factor, and oxytocin as indicators of equine welfare across three training regimens
Source: BMC Vet Res. 2026 Apr 30;22:355. doi: 10.1186/s12917-026-05507-7 (PMC13277048; doi:10.1186/s12917-026-05507-7)
Supplement: Supplementary file 1 — Supplementary Material 1. [file 12917_2026_5507_MOESM1_ESM.docx]

**Supplementary Tables**

**Supplementary Table 1 Baseline characteristics of horses by discipline.**

| **Discipline** | **Horses, n** | **Sex, n** | **Age, years** | **Breed, n** |
| --- | --- | --- | --- | --- |
| Show jumping | 17 | 9 mares, 8 geldings | 7.0 ± 3.1 (4–15) | KWPN (11); other (6)^1^ |
| Leisure | 14 | 9 mares, 5 geldings | 13.9 ± 5.7 (4–27) | other (14)^2^ |
| Race | 41 | 14 mares, 27 stallions | 3.3 ± 0.9 (2–7) | Arabian (29); Thoroughbred (12)^3^ |

Age is reported as mean ± SD (range). Breed categories are shown as recorded in the dataset. Sex categories are mares, geldings, and stallions.

¹ Show jumping breeds comprised Dutch Warmblood (KWPN; n = 11), Holsteiner (n = 1), Belgian Warmblood (n = 1), Polish Warmblood (n = 1), Anglo European Studbook (AES; n = 1), Małopolski (n = 1), and one horse of unknown breed (n = 1).
² Leisure breeds comprised Polish Warmblood (n = 3), Małopolski (n = 4), Fjord (n = 3), Arabian (n = 2), Felin Pony (n = 1), and one horse of unknown breed (n = 1).
³ Racehorse breeds comprised Arabians and Thoroughbreds; two horses did not contribute complete paired observations for any biomarker and were excluded from inferential analyses, yielding an analytic dataset of n = 41.

**Supplementary Table 2 Data completeness by discipline and marker.**

| **Discipline** | **Marker** | **Total records, n** | **Paired observations, n** | **Missing pre, n** | **Missing post, n** |
| --- | --- | --- | --- | --- | --- |
| Show jumping | Serotonin (5-HT) | 17 | 14 | 1 | 2 |
| Show jumping | BDNF | 17 | 15 | 0 | 2 |
| Show jumping | Cortisol | 17 | 15 | 0 | 2 |
| Show jumping | Oxytocin | 17 | 7 | 6 | 10 |
| Leisure | Serotonin (5-HT) | 14 | 12 | 1 | 1 |
| Leisure | BDNF | 14 | 12 | 1 | 1 |
| Leisure | Cortisol | 14 | 12 | 1 | 1 |
| Leisure | Oxytocin | 14 | 4 | 8 | 6 |
| Race | Serotonin (5-HT) | 113 | 109 | 2 | 4 |
| Race | BDNF | 113 | 104 | 6 | 6 |
| Race | Cortisol | 113 | 69 | 42 | 44 |
| Race | Oxytocin | 113 | 101 | 8 | 7 |

Total records represent horses for show jumping and leisure, and sessions for race. Paired observations indicate records with non-missing pre and post values for ln(post/pre).

**Supplementary Table 3 Post/pre ratios in show jumping and leisure horses (pooled within discipline).**

| **Discipline** | **Marker** | **Paired observations, n** | **Post/pre ratio (95% CI)** | **P value** |
| --- | --- | --- | --- | --- |
| Leisure | BDNF | 12 | 1.10 (0.64–1.89) | 0.714913 |
| Leisure | Cortisol | 12 | 0.96 (0.65–1.41) | 0.80863 |
| Leisure | Oxytocin | 4 | 0.98 (0.29–3.29) | 0.966703 |
| Leisure | Serotonin (5-HT) | 12 | 1.37 (0.86–2.19) | 0.166304 |
| Show jumping | BDNF | 15 | 1.28 (0.96–1.72) | 0.087514 |
| Show jumping | Cortisol | 15 | 0.95 (0.68–1.31) | 0.726692 |
| Show jumping | Oxytocin | 7 | 0.88 (0.71–1.09) | 0.194972 |
| Show jumping | Serotonin (5-HT) | 14 | 0.63 (0.49–0.82) | 0.002088 |

Post/pre ratios and 95% CIs were derived from models fitted on ln(post/pre), as described in Statistical analysis. 5-HT, 5-hydroxytryptamine; BDNF, brain-derived neurotrophic factor.

**Supplementary Table 4 Racehorses: mixed-effects model marginal mean post/pre ratios by session and sex.**

| **Marker** | **Session** | **Sex** | **Paired sessions, n** | **Post/pre ratio (95% CI)** | **P value** |
| --- | --- | --- | --- | --- | --- |
| Serotonin (5-HT) | T1 | Mare | 14 | 1.15 (0.76–1.75) | 0.498515 |
| Serotonin (5-HT) | T1 | Stallion | 28 | 1.31 (0.94–1.82) | 0.112358 |
| Serotonin (5-HT) | T2 | Mare | 13 | 1.17 (0.76–1.78) | 0.48169 |
| Serotonin (5-HT) | T2 | Stallion | 21 | 1.32 (0.91–1.91) | 0.139212 |
| Serotonin (5-HT) | Race day | Mare | 12 | 1.95 (1.26–3.02) | 0.002788 |
| Serotonin (5-HT) | Race day | Stallion | 21 | 2.21 (1.53–3.18) | 0.000024 |
| BDNF | T1 | Mare | 11 | 1.11 (0.71–1.71) | 0.655257 |
| BDNF | T1 | Stallion | 27 | 1.03 (0.73–1.44) | 0.874371 |
| BDNF | T2 | Mare | 11 | 1.01 (0.65–1.58) | 0.956002 |
| BDNF | T2 | Stallion | 21 | 0.94 (0.65–1.36) | 0.750371 |
| BDNF | Race day | Mare | 12 | 0.96 (0.62–1.49) | 0.859637 |
| BDNF | Race day | Stallion | 22 | 0.89 (0.62–1.28) | 0.543308 |
| Oxytocin | T1 | Mare | 12 | 0.88 (0.53–1.45) | 0.61791 |
| Oxytocin | T1 | Stallion | 23 | 0.87 (0.57–1.32) | 0.507058 |
| Oxytocin | T2 | Mare | 13 | 0.97 (0.59–1.59) | 0.897942 |
| Oxytocin | T2 | Stallion | 20 | 0.95 (0.61–1.49) | 0.834356 |
| Oxytocin | Race day | Mare | 11 | 0.97 (0.58–1.63) | 0.922229 |
| Oxytocin | Race day | Stallion | 22 | 0.96 (0.62–1.48) | 0.853628 |
| Cortisol | T1 | Mare | 11 | 1.06 (0.73–1.54) | 0.749448 |
| Cortisol | T1 | Stallion | 15 | 0.86 (0.61–1.20) | 0.373611 |
| Cortisol | T2 | Mare | 10 | 0.87 (0.59–1.28) | 0.482246 |
| Cortisol | T2 | Stallion | 12 | 0.70 (0.49–1.01) | 0.059486 |
| Cortisol | Race day | Mare | 8 | 0.88 (0.58–1.33) | 0.545537 |
| Cortisol | Race day | Stallion | 13 | 0.71 (0.50–1.02) | 0.064318 |

Marginal mean post/pre ratios and 95% CIs were derived from linear mixed-effects models fitted on ln(post/pre), with a random intercept for horse and fixed effects for session, sex, and breed, as described in Statistical analysis. Paired sessions indicate records with non-missing pre and post values for the respective marker.

**Supplementary Table 5 Show jumping horses: sex-stratified post/pre ratios (intercept-only models).**

| **Marker** | **Sex** | **Paired observations, n** | **Post/pre ratio (95% CI)** | **P value** |
| --- | --- | --- | --- | --- |
| Serotonin (5-HT) | Mare | 6 | 0.52 (0.30–0.88) | 0.025218 |
| Serotonin (5-HT) | Gelding | 8 | 0.74 (0.55–0.99) | 0.041964 |
| BDNF | Mare | 7 | 0.98 (0.68–1.42) | 0.893788 |
| BDNF | Gelding | 8 | 1.63 (1.05–2.52) | 0.034832 |
| Oxytocin | Mare | 4 | 0.91 (0.57–1.46) | 0.58233 |
| Oxytocin | Gelding | 3 | 0.84 (0.59–1.20) | 0.172148 |
| Cortisol | Mare | 7 | 0.83 (0.57–1.21) | 0.270031 |
| Cortisol | Gelding | 8 | 1.06 (0.58–1.94) | 0.82103 |

Sex-stratified estimates were derived from intercept-only models fitted on ln(post/pre) within each sex, as described in Statistical analysis.

**Supplementary Table 6 Show jumping horses: breed-adjusted sex-specific post/pre ratios.**

| **Marker** | **Sex** | **Paired observations, n** | **Post/pre ratio (95% CI)** | **P value** |
| --- | --- | --- | --- | --- |
| Serotonin (5-HT) | Mare | 14 | 0.47 (0.25–0.88) | 0.025695 |
| Serotonin (5-HT) | Gelding | 14 | 0.79 (0.47–1.34) | 0.320726 |
| BDNF | Mare | 15 | 0.91 (0.57–1.47) | 0.665416 |
| BDNF | Gelding | 15 | 1.73 (1.12–2.67) | 0.020908 |
| Oxytocin | Mare | 7 | 0.95 (0.57–1.59) | 0.451679 |
| Oxytocin | Gelding | 7 | 0.79 (0.42–1.48) | 0.131702 |
| Cortisol | Mare | 15 | 0.87 (0.42–1.76) | 0.646021 |
| Cortisol | Gelding | 15 | 1.03 (0.53–1.97) | 0.930953 |

Sex-specific marginal means were derived from OLS models fitted on ln(post/pre) with fixed effects for sex and breed, as described in Statistical analysis. Estimates were standardized over the observed breed distribution.

**Supplementary Table 7 Leisure horses: sex-stratified post/pre ratios (intercept-only models).**

| **Marker** | **Sex** | **Paired observations, n** | **Post/pre ratio (95% CI)** | **P value** |
| --- | --- | --- | --- | --- |
| Serotonin (5-HT) | Mare | 8 | 1.20 (0.74–1.96) | 0.401873 |
| Serotonin (5-HT) | Gelding | 4 | 1.78 (0.35–9.19) | 0.343136 |
| BDNF | Mare | 8 | 0.88 (0.41–1.85) | 0.68897 |
| BDNF | Gelding | 4 | 1.72 (0.61–4.83) | 0.193365 |
| Oxytocin | Mare | 2 | NA | NA |
| Oxytocin | Gelding | 2 | NA | NA |
| Cortisol | Mare | 8 | 0.87 (0.47–1.59) | 0.594116 |
| Cortisol | Gelding | 4 | 1.17 (0.78–1.74) | 0.306158 |

Sex-stratified estimates were derived from intercept-only models fitted on ln(post/pre) within each sex. Oxytocin could not be estimated within sex because n < 3.

**Supplementary Table 8 Racehorses: serotonin post/pre ratios by session and sex (OLS; no random effects).**

| **Session** | **Sex** | **Paired sessions, n** | **Post/pre ratio (95% CI)** | **P value** |
| --- | --- | --- | --- | --- |
| T1 | Mare | 14 | 0.96 (0.63–1.45) | 0.825411 |
| T1 | Stallion | 28 | 1.45 (1.14–1.84) | 0.004204 |
| T2 | Mare | 13 | 1.26 (0.83–1.89) | 0.24614 |
| T2 | Stallion | 21 | 1.28 (0.85–1.92) | 0.222984 |
| Race day | Mare | 12 | 2.43 (0.89–6.61) | 0.077465 |
| Race day | Stallion | 21 | 2.04 (1.11–3.72) | 0.023095 |

Supplementary subgroup OLS models were fitted on ln(post/pre) within each session-by-sex subgroup and do not account for within-horse clustering.

**Supplementary Table 9 Racehorses: pooled post/pre ratios by sex (OLS; no random effects).**

| **Marker** | **Sex** | **Paired sessions, n** | **Post/pre ratio (95% CI)** | **P value** |
| --- | --- | --- | --- | --- |
| Serotonin (5-HT) | Mare | 39 | 1.40 (0.98–1.99) | 0.064194 |
| Serotonin (5-HT) | Stallion | 70 | 1.54 (1.23–1.94) | 0.000336 |
| BDNF | Mare | 34 | 1.02 (0.66–1.55) | 0.940379 |
| BDNF | Stallion | 70 | 0.97 (0.79–1.18) | 0.725336 |
| Oxytocin | Mare | 36 | 0.95 (0.66–1.37) | 0.77412 |
| Oxytocin | Stallion | 65 | 0.92 (0.68–1.25) | 0.582489 |
| Cortisol | Mare | 29 | 0.95 (0.68–1.31) | 0.731304 |
| Cortisol | Stallion | 40 | 0.76 (0.60–0.96) | 0.022617 |

Supplementary pooled OLS models were fitted on ln(post/pre) within each sex subgroup across sessions and do not account for within-horse clustering.
